# A prospective non-randomized feasibility study of an online membership-based fitness program for promoting physical activity in people with mobility impairments

**DOI:** 10.1186/s40814-024-01528-x

**Published:** 2024-08-02

**Authors:** Laurie A. Malone, Tapan Mehta, Christen J. Mendonca, Sangeetha Mohanraj, Mohanraj Thirumalai

**Affiliations:** 1 Department of Occupational Therapy, School of Health Professions, The University of Alabama at Birmingham, Birmingham, AL USA; 2 Department of Family and Community Medicine, The University of Alabama at Birmingham, Birmingham, AL USA; 3 School of Health Professions, UAB Research Collaborative, The University of Alabama at Birmingham, Birmingham, AL USA; 4 Division of Preventive Medicine, The University of Alabama at Birmingham, Birmingham, AL USA

**Keywords:** Physical disability, Physical activity, Online fitness, Community program

## Abstract

**Background:**

People with mobility limitations have a disproportionately higher rate of acquiring secondary conditions such as obesity, cardiovascular comorbidity, pain, fatigue, depression, deconditioning, and type 2 diabetes. These conditions often result from poor access to home and community-based health promotion/wellness programs. The aim of this project was to determine the feasibility of delivering an online community membership-based fitness program for individuals with mobility impairments.

**Methods:**

For this prospective single-arm study, participants were recruited from members of a community fitness facility that serves people with physical disabilities and chronic health conditions. While all members had access to the online platform, individuals had to opt-in to participate in the research component. Activity options included 16 pre-recorded videos and 9 live exercise classes. During the 8-week program, participants had an opportunity to earn three exercise incentives for reaching certain activity milestones. Enrollment percentage, attendance, and attrition were tracked to assess program feasibility and acceptability. Changes in participant-reported outcomes including self-reported physical activity, psychosocial outcomes, and health-related quality of life (HRQOL) were examined using non-parametric analyses.

**Results:**

A total of 146 eligible individuals were screened of which 33 enrolled (22.6%). Two participants withdrew from the study, so a total of 31 were used for analyses. Participants included 29 women and 12 Black people with an average age of 60 (± 15.9) years. Health conditions included stroke, post-polio, arthritis, neuropathy, cerebral palsy, and obesity. Ten participants used an assistive device to get around inside the home. Twenty-six participants (78.8%) completed the online program, and 5 participants earned all 3 participation incentives. The mean number of live Zoom exercise classes attended by the participants was 12.8 (range = 0–43) over 8 weeks; 3 of 31 participants did not attend any classes. On average, participants watched 128 min (range = 0–704 min) of pre-recorded videos; 6 of 31 participants did not view any pre-recorded videos. Self-reported physical activity showed the largest improvement (11.15 units; 95% CI, 3.08, 19.56) with an effect size of 0.51 (Cohen’s d).

**Conclusions:**

This pilot study of an online membership-based fitness program for people with mobility impairments demonstrated preliminary effectiveness in increasing physical activity and was found to be feasible and acceptable. Feasibility endpoints do indicate potential to improve retention. These results suggest that online delivery of exercise programs can broaden the reach of specialized community fitness programs and is a promising direction for future work and fully powered trials are warranted to assess intervention efficacy.

**Trial registration:**

ClinicalTrials.gov, NCT05138809. Registered September 2, 2021, ClinicalTrials.gov PRS: Record Summary NCT05138809.

## Key messages regarding feasibility

- What uncertainties existed regarding the feasibility? The feasibility of implementing a completely online membership-based community exercise program for people with mobility limitations is unclear. Such a program has the potential to overcome certain barriers (i.e., transportation, cost) and broaden the reach of adaptive exercise programming to people with mobility limitation across the USA.
- What are the key feasibility findings? Delivery of an online membership-based community fitness program for people with mobility impairments is feasible and acceptable. Preliminary estimates from the pilot study indicate improvements in physical activity.
- What are the implications of the feasibility findings for the design of the main study? This pilot and feasibility study provides data to design a well-powered trial and revise the protocol to further enhance feasibility endpoints and assess other health-related outcomes.

## Background

Over the decades, studies conducted across the globe have collected substantiating evidence on the direct association between physical activity and its positive effects on health. Regular exercise has innumerable benefits on health regardless of age, gender, or ethnicity. Lack of adequate physical activity increases the risk of acquiring multiple chronic morbidities and disabilities and escalates the probability of death. Evidence demonstrates that regular physical activity reduces mortalities associated with stroke, diabetes mellitus, hypertension, coronary artery disease, and some forms of cancer [1]. According to a report published by CDC in January 2020 on the prevalence of physical inactivity among adults in the USA, the range of adult physical inactivity was estimated to be between 17.3 and 47.7% across all the states and territories [2].

A report published by CDC in September 2020 states that 61 million adults live with disability in the United States implying that 26% of adults (1 in 4 adults) in the country are affected with some type of disability [3]. Despite the knowledge about the benefits of physical activity, recreation, and structured exercise in promoting health and function in the general population, people with disability (PWD) are accounted to be one of the least active populations in society. The prevalence of inactivity among PWD was measured to be much higher as compared to people without disability [4]. This physical inactivity and resulting deconditioning may be associated with a variety of health deficits and increases the susceptibility of developing functional limitations; secondary health conditions (e.g., obesity, depression, or social isolation); and other co-morbidities among PWD [5].

The ability to start and continue a structured exercise program among PWD is often limited due to various secondary health conditions such as anxiety, depression, limited mobility, and pain, which in turn compromises their function and health. Since increasing physical activity and incorporating exercise into an individual’s daily routine is considered essential for the prevention and management of health conditions, professionals across healthcare domains are taking the initiative to encourage PWD to enroll/engage in routine exercise programming. However, in doing so, there are numerous challenges that have been identified across various levels of the socio-ecological model. A study published by Rimmer et al., on physical activity participation among PWD, identified various barriers to participation in fitness and recreational programs [6]. Some of the factors included natural and built environment, information barriers, availability of resources, and emotional and psychological barriers among many others.

The translation of evidence into practice (e.g., comprehensive care of PWD by healthcare providers) is a key factor underlying the successful adoption and maintenance of exercise behavior in PWD. Our recent qualitative research, for example, has indicated that people with multiple sclerosis want exercise recommendations from their healthcare providers [7]. These recommendations could include referrals to community fitness facilities. However, to address barriers, such as transportation and increasing the reach of exercise programs provided by community fitness facilities, an online virtual exercise platform is necessary. Furthermore, during the recent COVID-19 pandemic, online programming became important as on-site exercise options were limited for everyone as a precaution to reduce exposure by limiting social interactions and avoidance of close contact with other individuals. To address this concern, telehealth and other remote options became more prevalent. For more vulnerable groups, such as the elderly, stay-at-home orders were even more important, and an incentive to consider home-based exercise [8, 9]. Studies have begun to examine the feasibility of online physical activity programming in various groups including women over 50 years of age [10] and youth with disabilities [11]. In particular, a recent study found that a 4-week online physical activity program for youth with disabilities, based on the social cognitive theory, was feasible and acceptable [11].

The feasibility of implementing a completely online membership-based community exercise program for people with mobility limitations is unclear. Such a program has the potential to overcome certain barriers (i.e., transportation, cost) and broaden the reach of adaptive exercise programming to people with mobility limitation across the USA. The aim of this study was to assess the feasibility of delivering a virtual exercise program to members of a specialized community fitness facility and to examine participant acceptability of the program among members with mobility impairments. Enrollment percentage, attendance, and attrition were tracked to assess program feasibility and acceptability. Changes in participant-reported outcomes including self-reported physical activity, psychosocial outcomes, and health-related quality of life (HRQOL) were also examined.

## Methods

### Study setting eligibility criteria

**Fig. 1:**
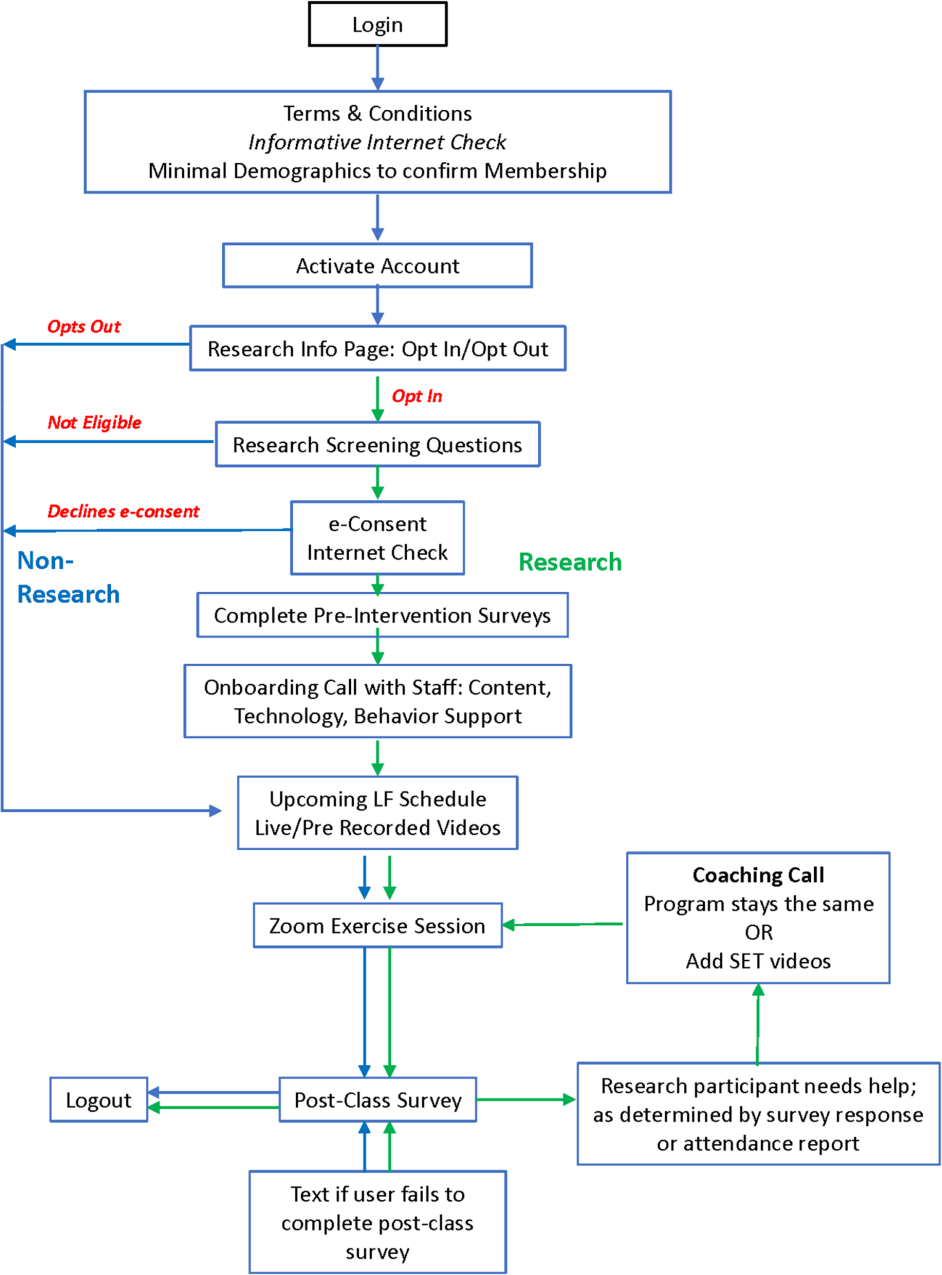
Overview of the Lakeshore online fitness program process

The inclusive virtual exercise system for community fitness centers was piloted at Lakeshore Foundation, a specialized fitness facility in Birmingham, AL, USA, that serves individuals with physical disabilities and chronic health conditions. Lakeshore delivers various physical activity, recreation, sport, and health promotion activities to qualifying individuals across the lifespan. All members aged 18 years or above were invited to enroll in the online virtual exercise system introduced and customized as the Lakeshore Online Fitness (LOF) program. The process for enrolling and participating in the program is shown in Fig. 1. The members visiting the LOF website were presented with a concise introduction (written and video) to the research component of LOF. They were then provided with the option to “Opt-In” to receive further information regarding the study. The members who opted in were screened based on inclusion (member of Lakeshore Foundation, age 18 years or above, mobility limitation, problem with gait, balance and/or coordination, fluency in English), and exclusion (no access to internet) criteria. Members who were screened as eligible for the study were asked to complete an e-consent form, followed by a survey packet to assess level of physical activity, reasons for exercise, self-efficacy for exercise, expectations for exercise, exercise goals and plans, and health-related quality of life (HRQOL). Participants who were not eligible for the study or declined to sign the e-consent continued to retain access to the LOF platform for regular access to the program options.

Eligible and consented participants were enrolled into an 8-week program and had access to 16 pre-recorded videos (asynchronous) and 9 live (synchronous) exercise classes each week on the online platform. With both asynchronous and synchronous modes available participants were encouraged to participate in exercise classes based on their preferences with a focus on adherence and obtaining a certain number of minutes each week. Participants were encouraged to utilize the platform several times per week increasing their minutes of activity each week.

### Description of the intervention

All online fitness classes were taught by the community center’s experienced fitness specialists, all with advanced training in inclusive adapted physical activity or exercise physiology. Instructors were skilled at designing, adapting, and delivering fitness classes to meet the needs of participants with various mobility impairments and ability levels.

The following 9 live classes were available to participants each week:

- Total body conditioning (Wed 1:00 pm; Thur 1:00 pm, Fri 1:00 pm)
- ABCs of balance (Mon 1:00 pm; Wed 9:45 am)
- Upper body basics (Mon 9:45 am; Fri 9:45 am)
- Seated yoga (Tues 1:00 pm; Thur 9:45)

In addition, during the program for the first 9 participants, a Zumba class was offered. However, this class was phased out by the fitness facility and no longer delivered remotely. After the end of each live class, participants were presented with a brief survey regarding their perceptions of the class. The pre-recorded videos were split into 3 playlists: yoga, balance, and upper body and core. Yoga had a series of 3 videos, balance 2 videos, and upper body and core 11 videos. The research team monitored participants’ weekly minutes of activity and post-survey responses, with coaching calls made at the end of weeks 2, 4, and 6 based on an issue being reported on the post-class survey during the previous 2 weeks or meeting of pre-determined online activity minutes criteria. For coaching call 1 (end of week 2)—less than 27 activity minutes for both weeks 1 and 2. For coaching call 2 (end of week 4)—less than 45 activity minutes for both weeks 3 and 4. For coaching call 3 (end of week 6)—less than 63 activity minutes for both weeks 5 and 6. Participants that expressed issues or lack of interest with the existing classes during the coaching call were provided with a new series of pre-recorded exercise videos for the subsequent 3 weeks. Each set included a series of videos for a complete routine: upper and lower body range of motion (i.e., stretching), aerobic routine, muscle strength training, functional strength and balance routine, and a cool down. Each series could be performed either seated or standing.

Based on conversations with the community partner, it was decided that small gift incentives would be provided to encourage participation along the way. Past experience of the community partner suggested that members appreciated small tokens of acknowledgement. The adherence incentives were based on the following progression: weeks 1–2: 90 min of LOF activity (pre-recorded or live) over the course of the 2 weeks; weeks 3–5: 225 min of LOF activity over the course of the 3 weeks; weeks 6–8: 315 min of LOF activity over the course of the 3 weeks. The gifts provided to participants at each stage were as follows: (1) yellow and red exercise resistance bands, (2) exercise disc sliders, and (3) insulated lunch bag.

At the end of the 8-week intervention, the participants were asked to complete the post intervention survey package. Compensation in the form of gift cards were given to the participants after completing pre- and post-assessments. An incentive in the form of a $25 gift card was given after the completion of baseline assessments and a $40 gift card after completing the post-assessments at the end of the 8 weeks. The protocol for this study is available at: https://clinicaltrials.gov/study/NCT05138809.

### Primary feasibility outcomes

A number of feasibility parameters were documented over the course of the program including enrollment percentage, attendance, and attrition. Given that participants who were not eligible for the study or declined to sign the e-consent still had access to the LOF platform as part of their membership, the enrollment target was set at 25%. The target for number of participants reaching the physical activity goal for each incentive period was set at 60% for the first incentive period (weeks 1–2), decreasing by 10% for each of the subsequent activity periods (weeks 3–5; weeks 6–8). Considering the short duration of the intervention, the expected dropout rate or attrition was set at 20%.

### Secondary participant-centered outcomes

Participant health history, physical activity, and psychosocial measures were assessed using the surveys indicated below.

#### Health history form (baseline only)

The health history form, completed by all members, was referred to during coaching calls to assist in planning physical activity goals for participants. In addition, it provided basic demographic information for use in describing the participant characteristics. The form consisted of a list of physical disabilities and chronic health conditions that qualify individuals for membership at the community facility. The list included disabilities related to heart, respiratory, neurological, and orthopedic conditions among many others. The survey also contained questions that were specific to certain health conditions including when and how the health condition was acquired and information regarding the type of condition. Participants were asked to fill out this form prior to the start of the 8-week intervention. The approximate time estimated to fill this form was 5 min.

#### Current level of physical activity

The Godin Leisure-Time Exercise Questionnaire is a 3-question survey that was developed to measure the average amount of strenuous, moderate, and mild/light leisure time exercise that a person engages in each week. Scoring produces a weekly leisure-time activity score with the following interpretation: active, 24 units or more; moderately active, 14 to 23 units; insufficiently active/sedentary, less than 14 units [12]. A new scoring method using only minutes of moderate and strenuous weekly activity was developed and recently validated among individuals with disabilities [12–14]. This method produces a Health Contribution Score (HCS) that aligns with physical activity recommendations. The HCS ranges from 0 to 98 units and is then converted to activity categories as used in previous scoring methods: active, HCS ≥ 24 units; moderately active, HCS 14 to 23 units; and insufficiently active, HCS < 14 units. This survey was completed by participants in ≤ 1 min.

#### Reasons for exercise

A Likert scale survey was developed by staff to understand the importance of various components of exercise for participants. A list of 10 components was included in the survey including balance, physical function, energy level, endurance, muscle strength, weight management, pain management, enjoyment, stress relief, and social engagement. The participant rated each component from 1 (not important) to 5 (very important) as to the importance of that component to them as a reason for exercise. The time for participants to complete the survey was estimated to be less than 1 min.

#### Self-efficacy for exercise scale

The Self-efficacy for Exercise (SEE) Scale was designed to help understand the level of confidence an individual has to independently exercise 3 times per week for 20 min under various conditions (i.e., weather, boredom, pain). The SEE scale consists of a series of 9 survey questions with a Likert scale format from 0 (not confident) to 10 (very confident). The responses to each question are summed for a total score ranging from 0 to 90. A higher score indicates higher self-efficacy for exercise. Testing has indicated the tool to be a reliable and valid measure of exercise self-efficacy [15]. Time for participants to complete the survey was estimated to be less than 1 min.

#### Multidimensional Outcome Expectations for Exercise Scale (MOESS)

This 15-question survey was designed to capture the beliefs and expectations of an individual regarding the multidimensional benefits of regular exercise or physical activity. Each question is a statement reflecting a possible benefit of exercise (e.g., “Exercise will improve my overall body functioning.”) Participants are asked to rate each statement on a scale from 1 = strongly disagree to 5 = strongly agree. The total score is the sum of all item ratings with a higher score indicating higher expectation for the outcome of the exercise. This tool has been reported to be a reliable and valid measure of outcome expectations for exercise in various populations [16–18]. This survey was estimated to be completed in 2 min.

#### Exercise goal-setting scale

This survey comprised of ten questions was designed to understand an individual’s ideology on how they set exercise goals and plan exercise activities [19]. Participants are asked to rate how well each statement describes them on a 5-point scale from 1 = does not describe to 5 = describes completely. This survey was estimated to be completed in 2 min.

#### Exercise planning/Scheduling scale

This survey was comprised of ten questions and was developed to understand an individual’s perspective on including exercise in their daily routine [19]. Participants were asked to rate the extent to which each statement describes them on a 5-point scale from 1 = does not describe to 5 = describes completely. This survey was estimated to be completed in 2 min.

#### Health-Related Quality of Life (SF36-E)

This survey was used to understand how participants interpreted their health as it relates to their quality of life. The information collected via the survey provided an indication of how participants felt and how well they were able to do their usual activities. The enabled version of the SF36 revised specific questions of the original survey to improve assessment among people with mobility impairments. Specifically, the words “walk” and “climb” were replaced with “go” and the stem of the physical function questions included the use of assistive devices [20, 21]. Scoring of the survey yields eight subscale scores specifically related to physical function, role limitations due to physical health, bodily pain, general health, vitality, social functioning, role limitations due to emotional problems, and mental well-being. In addition, two summary scores are produced including the physical component score and the mental component score [22]. The 36-item survey was estimated to be completed by participants in approximately 5 min.

We also tracked process, resource, and management outcomes such as the number and type of contact staff requests that the research team had to handle, LOF video analytics, attendance of sessions, and incentives distributed (Table 1).

**Table 1:** Key feasibility metrics, objectives, and question types

Metric	Objectives	Question types
**Feasibility outcomes**		
Process	Recruitment rate	Percent of individuals who follow through during the enrollment procedure
	Refusal rates	Frequencies and reasons for refusal and/or barriers experienced by members choosing not to participate in the study
	Attrition and retention rates	Frequencies and percentages of participant attrition and retention
Resource	Communication with participants	Frequency and time for communication between members and study staff to address operational issues
Management	Data collection and data entry	Time spent for outcome and system assessments
	Adverse events	Number of self-reported events by participants via system messaging, email, or telephone
**Secondary participant-centered outcomes**		
Scientific outcomes	Acceptability	Post-class surveys
	Pre-post changes	Physical activity and various psychosocial outcome surveys completed before and after the intervention

### Data analyses

Data analyses were conducted using R (4.1.2). Data were examined for errors, missingness, and outliers. Process, resource, administrative/management, and feasibility outcomes were examined via descriptive analyses. Feasibility was evaluated as the proportion of participants who completed the 8-week program. Participant sample characteristics were summarized using descriptive statistics. Participants were categorized into insufficiently active, moderately active, and active using Godin total and the health contribution scores [12–14]. Percentile bootstrap confidence intervals were estimated to draw inferences without assuming normality due to the relatively small sample size. In line with a pilot and feasibility study, we reported 75%, 85%, and 95% bootstrap confidence intervals to assess the changes between baseline and end of the 8-week program [23]. We descriptively analyzed the trends in exercise attendance and adherence using video watch time and Zoom class attendance data.

## Results

### Primary feasibility outcomes

#### Recruitment and retention

Participant characteristics are reported in Table 2, and an overview of the process for enrollment is represented in Fig. 1. Outreach to all Lakeshore Foundation members was done during the pre-determined time period of January to November 2021. This outreach was done by the membership team through email, newsletter, and website communications during the COVID-19 period when the facility was not open for in-person activities.

**Table 2:** Participant characteristics at baseline (*n* = 31)

	Overall
**Age**	
Mean (SD)	60.0 (15.9)
Median [Min, Max]	63.0 [22.0, 87.0]
IQR	23.5
**Sex**	
Male	2 (6.5%)
Female	29 (93.5%)
**Race**	
Black	12 (38.7%)
Hispanic	1 (3.2%)
White	18 (58.1%)
**Level of physical activity based on Godin overall score**	
Insufficiently active (< 14)	12
Moderately active (14–23)	5
Active ( ≥24)	14
**Level of physical activity based on Godin health contribution score**	
Insufficiently active	17
Moderately active	3
Active	11

**Fig. 2:**
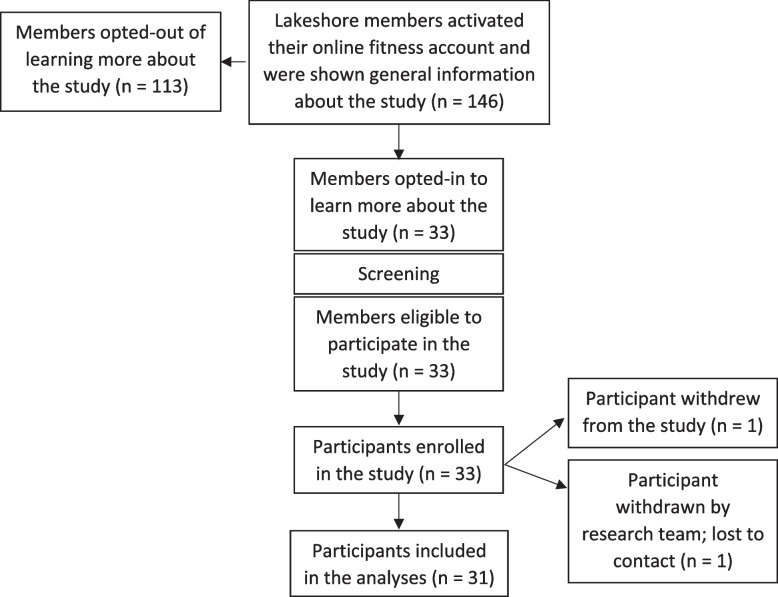
Flow of individuals through screening, enrollment, and data analyses

All active members of the Lakeshore Foundation community fitness center were invited. Out of 146 members who activated their online fitness account, 33 enrolled, consented, and completed baseline assessments (Fig. 2). The research team did not have access to information regarding individuals flowing through the membership system until they consented to the research study and therefore were unable to capture data regarding reasons for non-participation or lack of interest in the study. Members of the community center who did not opt-in to the research study still had access to the online fitness classes as part of their membership. Of the 33 who enrolled and completed the baseline assessments, one person did not participate in an onboarding call or any subsequent coaching calls, did not respond to email or voice messages, and did not engage in any online activity (< 10 min) during the 8 weeks and therefore was withdrawn and not included in the analyses. In addition, one person withdrew at the end of week 4. Out of the 31 participants included in the analyses, 26 completed the 8-week follow-up assessment. All participants were enrolled between September 2021 and March 2022. No adverse events were reported.

Participants included in the analyses were 94% (29/31) women and 39% (12/31) black people, with an average age of 60 ± 15.9 years (median age 63, range 22 to 87 years). Out of the 31 participants, 10 used one or more assistive devices to get around the house such as a cane (*n* = 5), walker (*n* = 3), manual or power wheelchair (*n* = 2), prosthetic leg (*n* = 1), or crutches (*n* = 1). Physical disabilities and chronic health conditions reported by the participants included arthritis (*n* = 21), obesity (*n* = 15), heart condition (*n* = 10), visual impairment (*n* = 10), neuropathy (*n* = 8), respiratory disease (*n* = 7), lymphedema (*n* = 5), diabetes (*n* = 5), cerebral palsy (*n* = 3), stroke (*n* = 3), lower limb amputation (*n* = 2; 1 lower leg, 1 foot), spinal cord injury (*n* = 1), epilepsy or seizure disorder (*n* = 1), and post-polio (*n* = 1). The use of an assistive device often depended on how the individual was feeling (e.g., leg pain). At baseline, 12 participants were considered to be insufficiently active as described by the Godin total weekly leisure-time activity overall score of < 14, 5 were moderately active (score 14 to 23), and 14 were classified as active based on a score ≥ 24 units [12]. Based on the HCS score, those who were insufficiently active to achieve health benefits increased to 17, with only 3 moderately active and 11 active (Table 2). The average Godin HCS was 17 (± 21) [13]. Looking at individual HCS scores, while 8 participants began the program active and remained active, 6 participants improved from inactive to active, and 3 went from moderately active to active. Seven participants remained inactive, and two participants changed from active to inactive.

#### Attendance

Out of the 31 participants, all but three (*n* = 28) attended at least one live session during the program. On average, participants attended 12.8 live sessions (range = 0 to 43) over the course of 8 weeks, with 6 participating in at least one live class each week. The average number of classes attended per week was 1.6, ranging from 0 to 9. Participation in live classes tended to decline over the 8-week period starting at an overall attendance of 56 in week 1 to 44 in week 8 (Table 3). The most popular class was total body conditioning followed by upper body basics. Eight participants continued to regularly (at least 4 times per month) attend the live classes for at least 2 months following completion of the 8-week program, with 5 participants continuing on for at least 6 months.

**Table 3:** Participation in live classes by week and by class

	**Week 1**	**Week 2**	**Week 3**	**Week 4**	**Week 5**	**Week 6**	**Week 7**	**Week 8**	**Total**
**ABCs of balance**	18	11	13	13	9	11	7	11	93
**Seated yoga**	15	13	9	4	9	9	9	6	74
**Total body conditioning**	17	19	14	16	15	12	9	11	113
**Upper body basics**	6	9	16	17	14	16	10	13	101
**Zumba**^a^	0	3	2	2	4	2	0	3	16
**All classes**	56	55	54	52	51	50	35	44	397

^a^Only offered during the program period for the first 9 participants

Twenty participants met the first incentive criteria of 90 min of exercise by the end of week 2, 12 met the second incentive criteria of 225 min of exercise during weeks 3 to 5, and 7 met the criteria of 315 min of exercise during weeks 6 to 8. Five participants received all three participation incentives, while 8 participants failed to meet any of the three exercise incentive criteria. Overall, 39 incentives were distributed to the 31 participants.

Each participant received up to three coaching calls to improve attendance and adherence. The first coaching call (CC1) was made at the end of week 2, for participants that engaged in less than 27 activity minutes each week for both weeks 1 and 2. A total of four participants qualified for CC1, and of those, two calls were completed. For each coaching call, three attempts were made. If the participant was not reached or did not return the phone call, that coaching session was marked as “did not complete.” The second coaching call (CC2) was conducted at the end of week 4 with those who had less than 45 activity minutes each week for both weeks 3 and 4. A total of 15 participants qualified for CC2, and of those, 12 calls were completed. The third coaching call (CC3) occurred at the end of week 6 with participants that had less than 63 activity minutes each week for both weeks 5 and 6. A total of 18 qualified for CC3, and of those 12 calls were completed. Per coaching call discussions, only one participant experienced difficulty or lack of interest with the existing classes and was provided with a supplementary 3-week series of pre-recorded exercise videos. Each week an exercise routine was provided, composed of a series of videos including warmup, aerobics, strength, balance, and cool-down.

#### Process and administrative outcomes

We also tracked process and administrative (resource, management) outcomes over the 8-week period (Table 1). The research team made 175 calls out of which 72 were onboarding calls, 81 calls were related to the coaching calls, 20 were related to surveys and two were miscellaneous. On average, each participant received five calls from the research team out of which two were answered and completed. The research team was contacted by the participants 56 times out of which 45 times was through email. Participants reached out to the research team for internet/technical issues (33), suggestions for videos (8), gifts/incentives (3), and two contacts related to surveys.

### Secondary participant-centered outcomes

In this section, the results of the post-class surveys and pre-post outcome measures are summarized.

#### Acceptability and preferences

Following synchronous classes, participants were asked to complete a short 5-question post-class evaluation survey. Table 4 indicates the reasons selected by the participants for joining a particular class. The most common reasons were “class time fits my schedule” and to “enhance my fitness/wellness.”

**Table 4:** Responses to the post-class survey question: “Why did you attend this class?”

Response choices	ABC’s of balance	Yoga	Total body conditioning	Upper body basics
Class is fun	6	5	6	2
Class time fits my schedule	10	13	13	8
Enhance my fitness/wellness	12	13	11	8
Instructor	4	5	5	2
It was recommended	1	1	2	1
Other	1	2	2	1

Participant acceptability of synchronous classes attended is reported in Table 5.

**Table 5:** Descriptive analyses of post-class acceptability survey items for synchronous classes

	**ABC’s of balance (Mon)**	**ABC’s of balance (Wed)**	**Seated yoga (Tue)**	**Seated yoga (Thu)**	**Total body conditioning (Wed)**		**Total body conditioning (Thu)**		**Total body conditioning (Fri)**		**Upper body basics (Mon)**		**Upper body basics (Fri)**		**Overall**	
	(*N* = 12)	(*N* = 20)	(*N* = 13)	(*N* = 18)	(*N* = 22)		(*N* = 19)		(*N* = 35)		(*N* = 31)		(*N* = 19)		(*N* = 189)	
**Q1 How much, if at all, did you enjoy the class? (1 = not at all, 5 = very much)**																
**Mean (SD)**	4.55 (0.820)	4.80 (0.696)	4.83 (0.577)	4.53 (0.915)		4.72 (0.958)		4.69 (0.704)		4.79 (0.620)		4.87 (0.346)		4.80 (0.561)		4.75 (0.674)
**Median [Min, Max]**	5.00 [3.00, 5.00]	5.00 [2.00, 5.00]	5.00 [3.00, 5.00]	5.00 [2.00, 5.00]		5.00 [1.00, 5.00]		5.00 [3.00, 5.00]		5.00 [2.00, 5.00]		5.00 [4.00, 5.00]		5.00 [3.00, 5.00]		5.00 [1.00, 5.00]
**Inter-quartile range (IQR)**	0.5	0	0	0.5		0		0		0		0		0		0
**Missing**	1 (8%)	0 (0%)	1 (8%)	3 (17%)		4 (18%)		3 (16%)		6 (17%)		1 (3%)		4 (21%)		23 (12%)
**Q2 How hard, if at all, do you feel like you were exercising during the class? (1 = not hard at all, 5 = very hard)**																
**Mean (SD)**	3.36 (0.674)	3.30 (1.22)	3.91 (1.04)	3.56 (1.09)		3.58 (1.12)		4.00 (0.894)		3.79 (0.995)		3.93 (1.18)		3.59 (0.870)		3.69 (1.05)
**Median [Min, Max]**	3.00 [2.00, 4.00]	3.00 [1.00, 5.00]	4.00 [2.00, 5.00]	3.50 [2.00, 5.00]		3.00 [1.00, 5.00]		4.00 [3.00, 5.00]		4.00 [2.00, 5.00]		4.00 [2.00, 5.00]		3.00 [2.00, 5.00]		4.00 [1.00, 5.00]
**IQR**	1	1.5	2	1.25		1.5		2		2		2		1		2
**Missing**	1 (8%)	0 (0%)	2 (15.%)	2 (11.%)		3 (14%)		3 (16%)		7 (20%)		3 (10%)		2 (11%)		23 (12%)
**Q3 How satisfied, if at all, were you with the instructor? (1 = not at all satisfied, 5 = very satisfied)**																
**Mean (SD)**	4.73 (0.647)	4.75 (0.716)	5.00 (0)	4.73 (0.704)		4.76 (0.970)		4.56 (0.814)		4.72 (0.702)		4.97 (0.183)		4.93 (0.258)		4.80 (0.627)
**Median [Min, Max]**	5.00 [3.00, 5.00]	5.00 [2.00, 5.00]	5.00 [5.00, 5.00]	5.00 [3.00, 5.00]		5.00 [1.00, 5.00]		5.00 [3.00, 5.00]		5.00 [2.00, 5.00]		5.00 [4.00, 5.00]		5.00 [4.00, 5.00]		5.00 [1.00, 5.00]
**IQR**	0	0	0	0		0		0.25		0		0		0		0
**Missing**	1 (8%)	0 (0%)	1 (8%)	3 (17%)		5 (23%)		3 (16%)		6 (17%)		1 (3%)		4 (21%)		24 (13%)
**Q4 Did you have difficulty completing the entire class due to issues with the exercise movements, pain, fatigue, or feeling unsafe? (yes or no)**																
**No**	10 (83%)	20 (100%)	11 (85%)	17 (94%)		18 (82%)		13 (68%)		26 (74%)		23 (74%)		18 (95%)		156 (83%)
**Yes**	2 (17%)	0 (0%)	0 (0%)	0 (0%)		1 (5%)		1 (5%)		2 (6%)		3 (10%)		0 (0%)		9 (5%)
**Missing**	0 (0%)	0 (0%)	2 (15%)	1 (6%)		3 (14%)		5 (26%)		7 (20%)		5 (16%)		1 (5%)		24 (13%)

In addition, as part of the pre-post intervention survey packet, participants were asked to select from a list of reasons to exercise, those that applied to them. The list included balance, endurance, pain management, enjoyment, muscle strength, social engagement, stress relief, physical functioning, and weight management. Among all the reasons presented, the majority of the participants reported physical functioning and balance improvement as the most important reasons for exercise (and joining the fitness facility). Most of the participants rated social engagement to be the least important reason for exercising.

### Preliminary effects

In line with statistical interpretation of pilot and feasibility studies, which are not powered for a confirmatory trial, we present 95%, 85%, and 75% percentile bootstrap confidence intervals based on the pre-post paired comparisons (Table 6). In terms of the evidence, we interpret findings at 95% confidence intervals to be high, 85% to be medium, and 75% to be minimally sufficient. We found that the self-reported physical activity (overall and health contribution scores) both increased. The overall score, which accounts for all types of physical activity, increased by 11.15 (95% CI, 3.08, 19.54) with an effect size of 0.51 (Cohen’s d). The Godin health contribution score for physical activity, which is linked with health-enhancing exercise, increased by 8.27 (95% CI, 0.08, 16.5) with an effect size of 0.38 These differences were found to be robust in sensitivity analyses. We also found that the SF36-E subdomain of general health improved at the 85% CI, while the evidence for improvement in physical functioning and energy/fatigue components was minimally sufficient at the 75% confidence interval. Our results also indicated that the SF36-E subdomain scores for role limitations due to emotional problems and social functioning decreased by 11.5 and 9 units with evidence level at the 75% confidence level.

**Table 6:** Results of the pre and post comparisons with paired bootstrap *t*-test

Variables	Mean (SD) pre	Mean (SD) post	Mean of differences	95% CI	85% CI	75% CI	Cohens d
**Godin Leisure-Time Exercise Questionnaire**							
Godin overall	33.03 (48.81)	38.08 (23.66)	11.15	(3.08, 19.54)	(5.19, 17.27)	(6.35, 16)	0.51
Godin health contribution	16.87 (21.06)	26.77 (21.94)	8.27	(0.08, 16.5)	(2.23, 14.31)	(3.46, 13.12)	0.38
Godin restricted on 7	25 (21.18)	37.27 (21.59)	10.35	(2.81, 17.85)	(4.81, 15.85)	(5.92, 14.77)	0.52
**Self-Efficacy for Exercise Scale**	46.68 (21.05)	44.5 (19.37)	− 4.27	(− 12.23, 3.08)	(− 10, 1.23)	(− 8.81, 0.19)	0.21
**Multidimensional Outcome Expectations for Exercise Scale (MOESS)**							
MOESS physical	27.87 (2.17)	27.77 (3.01)	− 0.12	(− 1, 0.77)	(− 0.77, 0.54)	(− 0.62, 0.38)	0.05
MOESS social	10.32 (4.17)	9.92 (4.73)	− 0.46	(− 1.69, 0.85)	(− 1.38, 0.5)	(− 1.19, 0.27)	0.14
MOESS self-evaluative	21.32 (2.89)	21.58 (3.75)	0.15	(− 1.04, 1.35)	(− 0.73, 1.04)	(− 0.54, 0.85)	0.05
MOESS Total	59.52 (6.89)	59.27 (9.55)	− 0.42	(− 2.73, 1.77)	(− 2.12, 1.23)	(− 1.77, 0.88)	0.07
**Exercise Goal Setting and Exercise Planning/Scheduling Scales**							
Exercise plans	29.35 (7.96)	30.27 (7.04)	1.12	(− 0.92, 3.23)	(− 0.42, 2.65)	(− 0.12, 2.35)	0.20
Exercise goals	28.39 (9.8)	27.81 (10.8)	− 0.92	(− 4.38, 2.73)	(− 3.5, 1.73)	(− 3, 1.15)	0.10
**Health-Related Quality of Life (SF36-E)**							
SF36-E physical functioning	47.74 (22.39)	52.5 (28.89)	5.19	(− 2.12, 12.69)	(− 0.19, 10.77)	(0.77, 9.62)	0.26
SF36-E role limitations due to physical health	51.61 (37.04)	51.92 (42.38)	− 1.92	(− 22.12, 18.27)	(− 16.35, 12.5)	(− 13.46, 9.62)	0.04
SF36-E role limitations due to emotional problems	72.04 (37.61)	60.26 (45.23)	− 11.54	(− 29.49, 6.41)	(− 24.36, 1.28)	(− 21.79, − 1.28)	0.24
SF36-E energy fatigue	48.06 (17.87)	54.62 (18.33)	4.62	(− 1.92, 10.77)	(− 0.19, 9.04)	(0.96, 8.27)	0.28
SF36-E emotional well being	72.65 (17.53)	74.46 (17.49)	1.23	(− 1.85, 4.46)	(− 1.08, 3.54)	(− 0.62, 3.08)	0.15
SF36-E social functioning	78.23 (23.04)	71.15 (29.32)	− 9.13	(− 19.71, 0)	(− 16.83, − 1.92)	(− 14.9, − 3.37)	0.34
SF36-E pain	60.16 (20.72)	64.13 (25.17)	1.92	(− 3.75, 7.4)	(− 2.21, 5.96)	(− 1.35, 5.19)	0.13
SF36-E general health	50.97 (18.46)	55 (17.03)	3.65	(− 0.19, 7.5)	(0.77, 6.54)	(1.35, 5.96)	0.36

## Discussion

We piloted an inclusive online community-based fitness program through a single-arm non-randomized trial. The objective was to assess feasibility, acceptability, and preliminary effect of delivering exercise online through a real-world implementation by partnering with a premier inclusive fitness facility. Implementation of the LOF program was done during the COVID-19 period when the members had limited or no access to in-person facility.

Approximately 22% of the members who accessed the LOF system decided to join the program. We observed that over 80% of participants who joined LOF were retained. The LOF participants included predominantly older women, and the majority of the participants were sedentary or not engaged in a sufficient amount of physical activity. Participants included African Americans and people with disabilities using assistive devices. Consideration should be given to program elements that could entice a wider range of demographics or be designed to target specific groups (i.e., men, younger adults with mobility impairments).

While over half of the participants completed a sufficient number of physical activity minutes to receive the first incentive at the end of the second week, this number declined over the weeks, which is a fairly common challenge in physical activity literature. A few factors may have influenced the number of people who engaged in a sufficient number of minutes each week to receive an incentive. First, over the course of the study, a few participants reported being sick or traveling thereby reducing their attendance record. Second, at the beginning of the project period, a few technical glitches were experienced not allowing participants to access the online classes. Such issues were typically resolved within 24 h but may not have been reported to the research team by a participant in time for a particular class. In addition, engagement in a physical activity done outside of the LOF program was not tracked and may have influenced attendance.

A closer look at the incentive data, however, reveals that many participants were close to reaching the recommended minutes of activity for each of the three-goal periods. For the first incentive, while 20 participants met the physical activity goal, an additional 5 participants reached over 60% of the goal. For the second incentive, while 12 participants reached the physical activity goal, an additional 4 participants reached over 60%, with two of those over 80% of the goal. Finally, for the third incentive, on top of the 7 participants who reached the physical activity goal, an additional 6 participants reached over 60%, with three of those over 80% of goal. Although the influence of the incentives was not directly examined, consideration must be given to their potential influence on participation and taken into account for planning and future delivery of such a program.

Coaching calls were a useful mechanism to assist participants in meeting the targets or retaining them in the program. Although it is difficult to track the influence of coaching calls on engagement with the program most participants expressed appreciation for the encouragement and personal contact by staff. A limitation of the coaching calls might have been related to timing, i.e., were the calls received when the participants still had time to increase their weekly minutes during a particular goal period. Unfortunately, coaching calls were often delayed as a result of staff scheduling, and the need for multiple voicemails and call-backs. A possible supplement to the coaching calls for increasing attendance in future studies would be to consider the inclusion of weekly prompts to participants using an ecological momentary assessment technique.

From a patient-centeredness point of view, it seems the primary motivation of the majority of LOF participants was to increase balance and physical functioning. The majority of participants did not rate emotional or social factors as the most important reason to engage in exercises through LOF.

Our preliminary findings were in line with the patient-centered motivators for exercise. In terms of preliminary effectiveness, evidence participating in LOF seems to have improved physical activity with some moderate evidence of improvement in physical functioning and energy. On the other hand, we observed a decline in the emotional health and social functioning component at the end of 8 weeks. There were no changes in the psychosocial outcomes even at the 75% confidence interval.

Members of the specialized community facility have traditionally been offered in-person exercises at their state-of-the-art facility. The LOF program was a rapid response due to the dramatic health risks and restrictions due to COVID-19. Overall, we confirmed the feasibility of the online inclusive community-based fitness platform through retention and the process outcomes. Qualitative interviews and post-class survey assessments confirmed the acceptability. We also found strong evidence suggesting that participating through this online platform improved physical activity. However, we also observed few challenges that could be addressed moving forward. We found the reach of the platform, defined as the number of individual members who accessed LOF and enrolled, to be limited. Yet, compared to other estimates, our reach in terms of percentage is superior [24]. We did not observe an improvement in the psychosocial outcomes and observed a decline in social functioning and emotional health domains. This aspect was particularly challenging since our members until COVID-19 were primarily geared towards in-person classes available at a state-of-the-art facility that promotes social engagement. Declines in these components could also be a factor of the stresses and restrictions during the COVID-19 time that our platform could not fully address. Further, the quick turnaround time in developing the platform [25] and launching the LOF program limited our ability to couple behavioral health coaching to enhance psychosocial and social functioning outcomes. Our coaching calls were triggered to address attendance and adherence rather than based on tracking social engagement.

Finally, our study had a few apparent limitations. We conducted a real-world implementation using a single-arm study that lacked a control arm. Hence, it is plausible we could have observed worse outcomes in a control group, and relative improvements in emotional health and social functioning may have been a function of the study being conducted during multiple COVID-19 waves. Our study was limited to assessing feasibility and findings are not generalizable since we partnered with a local specialized community fitness facility. Participants who were recruited were current members of the community center and therefore may have already been motivated to be physically active. Furthermore, the low enrollment rate may have been due to the fact that those who did not consent to the research study still had access to the online fitness classes as part of their membership. Finally, our study assessed only short-term immediate 8-week outcomes using self-reported surveys and no objective assessments.

## Conclusions

This current study describes the feasibility, acceptability, and potential effectiveness of delivering an online 8-week membership-based community fitness program for promoting physical activity among people with mobility impairments. Additional study is needed to increase generalizability to other participant groups, communities, and fitness centers. Considerations for future programming are to incorporate other intervention components beyond exercise such as behavioral coaching tailored for the participants that have already been demonstrated to be feasible and efficacious. Such components could be combined to develop more precise and tailored exercise interventions and then tested using optimization designs such as sequential multiple assigned randomized trial (SMART) and subsequent confirmatory trials.

## Data Availability

The datasets used and/or analyzed during the current study are available from the corresponding author on reasonable request.

## Abbreviations

CC1: First coaching call
CC2: Second coaching call
CC3: Third coaching call
HCS: Health contribution score
IQR: Interquartile range
HRQOL: Health-related quality of life
PWD: People with disability
SEE: Self-efficacy for exercise
LOF: Lakeshore Online Fitness Program
