# Correction: A prospective non‑randomized feasibility study of an online membership‑based fitness program for promoting physical activity in people with mobility impairments

**DOI:** 10.1186/s40814-024-01548-7

**Published:** 2024-09-20

**Authors:** Laurie A. Malone, Tapan Mehta, Christen J. Mendonca, Sangeetha Mohanraj, Mohanraj Thirumalai

**Affiliations:** 1 Department of Occupational Therapy, School of Health Professions, The University of Alabama at Birmingham, Birmingham, AL USA; 2 Department of Family and Community Medicine, The University of Alabama at Birmingham, Birmingham, AL USA; 3 School of Health Professions, UAB Research Collaborative, The University of Alabama at Birmingham, Birmingham, AL USA; 4 Division of Preventive Medicine, The University of Alabama at Birmingham, Birmingham, AL USA

**Correction: Pilot Feasib Stud 10, 104 (2024)**

**https://doi.org/10.1186/s40814-024-01528-x**

Following publication of the original article [1], the authors reported an error found under the headings Discussion, Acknowledgements and Authors' Contributions.

The updated Discussion, Acknowledgements and Authors' Contributions are given below and the changes have been highlighted in **bold typeface**.

**New reference added under Discussion (8th paragraph)**

… Declines in these components could also be a factor of the stresses and restrictions during the COVID-19 time that our platform could not fully address. Further, the quick turnaround time in developing the platform **[25]**…

 “25 Mohanraj S, Malone LA, Mendonca CJ, Thirumalai M. Development and formative evaluation of a virtual exercise platform for a community fitness center serving individuals with physical disabilities: Mixed methods study. JMIR Form Res. 2023;7:e49685. doi: 10.2196/49685.”

**Acknowledgements**

The authors are grateful for the assistance of **Crystal Russell, Amy Belcher, Carol Kutik, Jen Allred, and the fitness instructors from Lakeshore Foundation** for their contributions during recruitment and program delivery. The authors would also like to thank Venkat Raparla, **Kireeti Boddupali**, and other members of the ICT team for the design and development of the system. The authors are also grateful for the contributions of Vasil Bachiashvili for his assistance with data analyses.

**Authors’ contributions**

MT was responsible for the development of the **system, SM and MT were responsible for platform testing and protocol development**. LM and CM managed the delivery of the program. TM oversaw the analysis and interpretation of the data. TM and LM were major contributors in writing the manuscript. All authors read and approved the final manuscript.

The original article [1] has been updated.
